# Introducing 3D printed models of fractures in osteology learning improves clinical reasoning skills among first-year medical students: a pilot study

**DOI:** 10.1186/s12909-025-06746-2

**Published:** 2025-02-06

**Authors:** Abhishek Agarwal, Anne D Souza, B. Jyostna, Ashwija Shetty, Nandini Bhat

**Affiliations:** 1 Kasturba Medical College, Manipal, Manipal Academy of Higher Education, Manipal, India; 2 Department of Anatomy, Kasturba Medical College, Manipal, Manipal Academy of Higher Education, Manipal, India

**Keywords:** 3D printed models, Osteology, Intervention, Learning, Clinical reasoning, Anatomy

## Abstract

**Background:**

The human bone anatomy is commonly taught using normal adult bones. However, students often face difficulties comprehending the clinical correlations related to fractures, as they only rely on text content or diagrams without three-dimensional visual aids. Therefore, this study aims to evaluate the effectiveness of using 3D-printed models of limb bone fractures in routine osteology classes to enhance the clinical reasoning skills of first-year medical undergraduate students.

**Methodology:**

In this experimental study, 105 first-year medical undergraduate students were divided into intervention and control groups based on their pre-assigned serial numbers. The control group was taught using dry adult human bones, with the teacher explaining clinical correlations verbally. Meanwhile, in two sessions, the intervention group was taught using 3D-printed models of fractures in addition to real bones. At the end of the second session, students were evaluated for their clinical reasoning ability using a case-based MCQ test (maximum score 5). The scores were compared between the two groups using an unpaired t-test. Students of the intervention group were asked to rate their learning experience using a 10-point Likert Scale questionnaire.

**Results:**

The intervention group scored significantly higher (2.54 ± 1.15) than the control group (2.04 ± 0.94) (*p* = 0.015). The maximum score for both groups was five, and the minimum was one. Most students agreed that the 3D-printed models helped them understand the fractures’ clinical relevance and provided better orientation to the bones, joints, and structures involved in fractures (92%, *n* = 46). The students expressed a desire for more similar types of sessions.

**Conclusion:**

Incorporating 3D-printed models of fractures was a novel approach to help students comprehend the clinical correlations. This strategy improved students’ clinical reasoning skills in the intervention group, as evidenced by their higher scores and feedback. Therefore, 3D-printed models are a valuable addition to the traditional teaching methods of learning osteology.

**Supplementary Information:**

The online version contains supplementary material available at 10.1186/s12909-025-06746-2.

## Background

Anatomy education in the first year of a medical undergraduate program lays the foundation for a student’s understanding of the human body [1]. Traditionally, cadaveric teaching has been used, and it is still considered the gold standard for anatomy demonstration [2]. Though skeletal anatomy is routinely taught using normal real human bones, several advanced approaches, such as video-based and virtual models, have been practiced and proven effective [3, 4]. Clinical correlations related to the bones can be learned effectively using 2D tools such as X-ray or CT images or a more advanced approach of 3D printing, which has additional educational benefits [5].

The introduction of a competency-based curriculum in India has broadened the range of learning abilities of medical undergraduates by incorporating clinical reasoning skills through modules such as early clinical exposure and basic science correlation [6]. In this regard, a more focused learning approach towards clinical correlations would be required from the early professional years. Literature shows that a clinically integrated undergraduate course can potentially foster students’ early clinical reasoning skills [7, 8]. These skills can be assessed using the application and analysis levels of Bloom’s Taxonomy [9].

With the advance of technology, 3D printing has been used to develop real-life-like models of viscera, bones, and tissues with a wide range of implications [10]. 3D models are easy to create and can be utilized as a learning resource in anatomy in class and for self-study [11]. As a valuable resource for osteology learning, 3D-printed bones can be a remarkable alternative to real human bones in terms of accuracy and durability [12]. Additionally, if the students were allowed to develop these models, they would be encouraged to engage in active, personalized, and kinaesthetic learning [13]. Incorporating 3D printed models for osteology would also minimize the ethical concerns that emerge while procuring real human bones [14, 15].

Few researchers across universities have explored the impact of 3D printed models on the education of orthopedic residents in a clinical setting regarding improvement in understanding the morphology, surgeons planning the operation, and also to increase in patient’s and family’s satisfaction with an explanation of disease [16–19]. However, the utilization of 3D printing technology in understanding the clinical correlations of bones like fractures in anatomy education is explored minimally. Therefore, the present study examines the impact of using 3D-printed models in improving clinical reasoning among medical students.

In this regard, the present study was undertaken to incorporate 3D printed models of limb bone fractures (clavicle, humerus, femur, and tibia) during osteology classes for a better understanding of clinical correlations and thereby to improve clinical reasoning skills among first-year medical undergraduate students.

### Objectives

1. To determine the level of learner satisfaction in using 3D printed fracture models during the routine osteology classes.
2. To compare the learning outcomes between the traditional osteology classes (control group) and the classes with 3D printed fracture models (intervention group) at the end of the session.

## Materials and methods

### Description of existing osteology classes

As we follow a systemic approach, osteology is taught in the musculoskeletal system block. Osteology is taught to first-year medical undergraduates using a small group teaching (SGT) approach. Two hundred and fifty students of the entire batch are divided into groups of approximately 20 students. Each small group will have one anatomy staff assigned.

Real human bones, which are obtained from the departmental reserve, are utilized for learning. The students learn the basics, such as anatomical position and side determination, parts, and attachments. The relevant clinical correlations, such as fracture of a particular bone, are routinely discussed verbally. Each session may extend up to two hours.

### Study design and setting

This cross-sectional experimental study was conducted at the Anatomy Department of Kasturba Medical College, Manipal, India, in 2023. The sessions were conducted during January and February. The study was performed after approval from the Institutional Ethics Committee of Kasturba Hospital and Medical College, Manipal (IEC 1-348/2023). The study design was chosen as per the guidelines provided by Cook and Beckman by having a control group with a traditional learning approach [20].

As osteology learning occurs in the first year of medical school, using a convenient sampling method, one hundred and five first-year medical undergraduate students were included based on serial numbers. Based on serial numbers, students were further divided into the intervention group (*n* = 53) and the control group (*n* = 52). Written informed consent was taken from the students before the commencement of the study sessions. The students who were repeating first were excluded from the study, considering they had already been exposed to osteology previously.

**Fig. 1:**
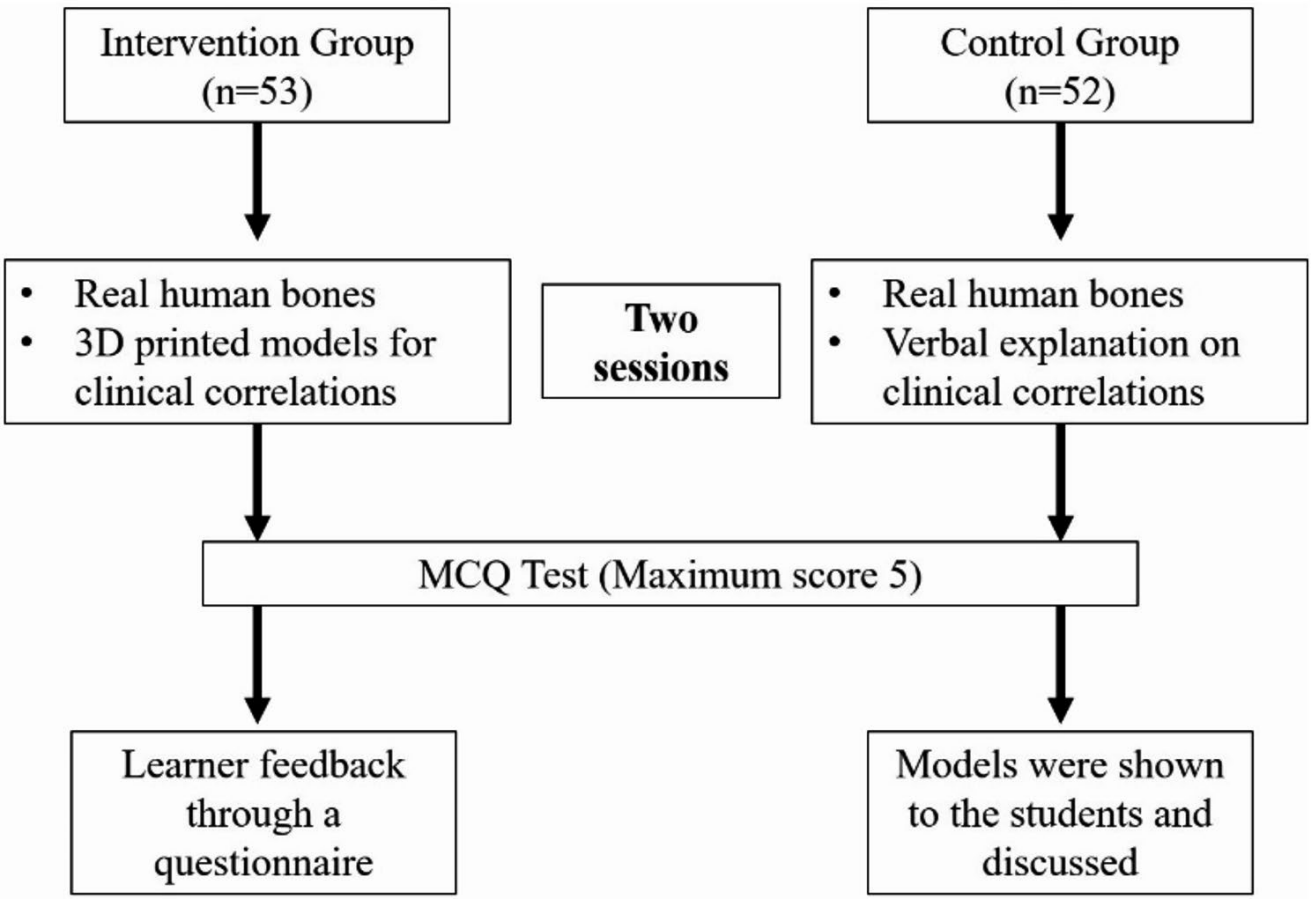
Flowchart explaining the study design. The flowchart describes a step-by-step approach to the intervention and the process of learning and assessment sessions

The students of the control group were taught osteology using real human bones. However, the intervention group was taught using the 3D-printed models of fractured bones in addition to the real human bones. One session was planned per week, with two such sessions completed in two weeks. A detailed description of the intervention is provided as a flow chart in Fig. 1. A detailed development process of the 3D models and the lesson plans is described below.

### Development of the 3D printed models

**Fig. 2:**
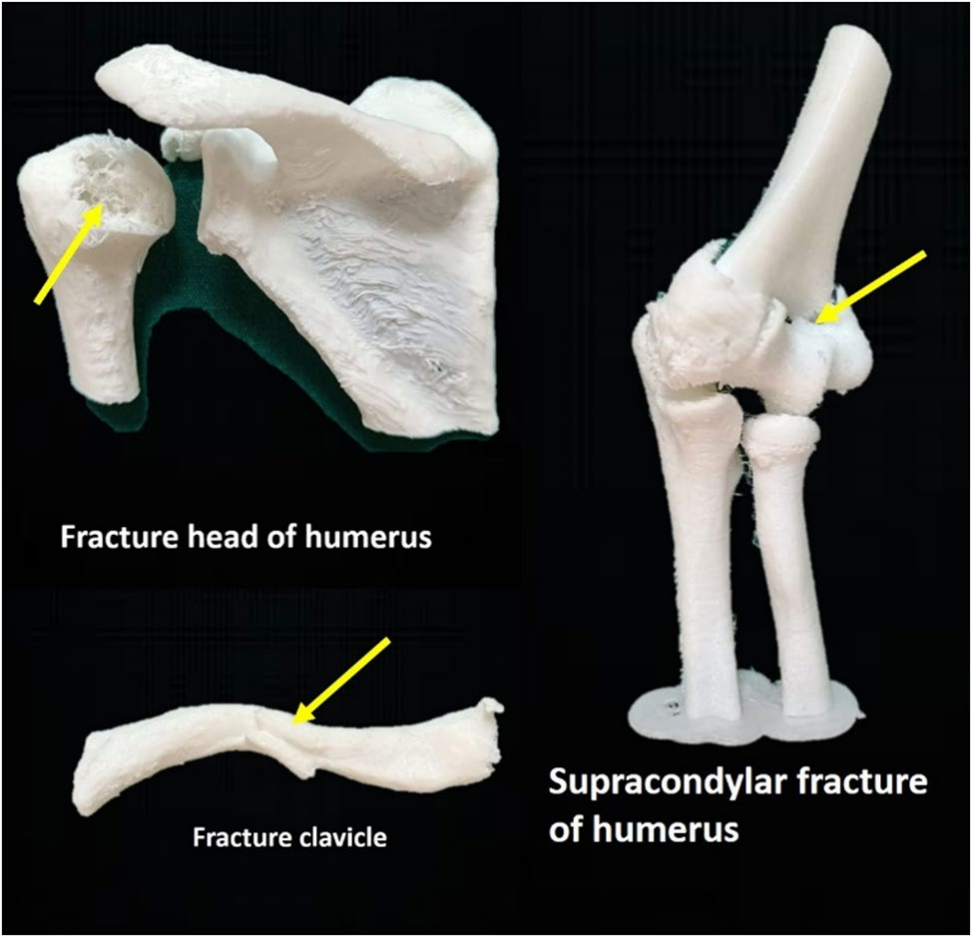
Photograph of the 3D-printed models of fractures of upper limb bones. The fractures are indicated using yellow arrows. The actual and printed dimensions of the models (in inches) are given below. Fracture head of the humerus (Actual size: Width 231×Height 180×Depth 141, Printed size: scaled to 5%). Fracture clavicle (Actual size: Width 190×Height 126×Depth 170, Printed size: scaled to 5%). Supracondylar fracture of humerus (Actual size: Width 49×Height 149×Depth 55, Printed size: scaled to 10%)

**Fig. 3:**
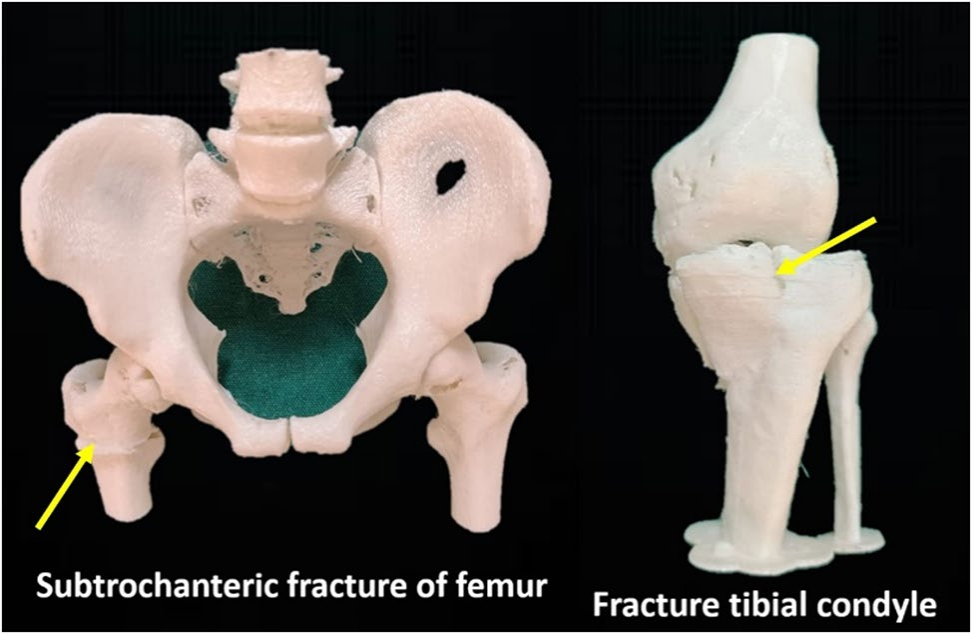
Photograph of the 3D-printed models of fractures of lower limb bones. The fractures are indicated using yellow arrows. The actual and printed dimensions of the models (in inches) are given below. Subtrochanteric fracture of femur (Actual size: Width 289×Height 261×Depth 142, Printed size: scaled to 5%). Fracture tibial condyle (Actual size: Width 101×Height 251×Depth 105, Printed size: scaled to 7%)

The models focussed on the fractures of the limb bones. We identified the clinical correlations to be addressed based on the list of competencies to be covered and created an inclusive list of competencies [21]. We chose five models of limb fractures; those were of the shoulder with proximal humerus fracture, fracture clavicle, elbow with supracondylar fracture of the humerus, hip joint with subtrochanteric femur fracture, a knee joint with fracture of the patella and medial condyle of tibia (Figs. 2 and 3). The above fractures’ Stereolithography (STL) files were obtained from the open-source exchange website https://3dprint.nih.gov/. The STL files were printed using the ‘Flashforge-Inventor’ 3D Printer (Zhejiang Flashforge 3D Technology Co., Ltd, China) housed in the Department of Anatomy, KMC Manipal. It was a Fused Deposition Modeling (FDM) type of printer with a nozzle diameter of 0.4 mm. The models were printed using Acrylonitrile Butadiene Styrene (ABS) and flexible materials.

### Development of a lesson plan

A team of faculty involved in osteology teaching developed lesson plans for the classes utilizing 3D printed models. One sample lesson plan is provided as Additional File 1. The plan was focused on the selected competencies. We used a reference model given by Anderson et al. to teach clinical reasoning in anatomy and developed our lesson plans [7]. The lesson plan emphasized a five-minute brainstorming- Introduction and identification of the model followed by a 15-minute discussion- the students will be asked to identify the site of the fracture, mention the possible anatomical structures affected, and discuss relevant clinical features, and the last 10 min take home message- used to revise and conclude the session. The lesson plans were validated for content by three Anatomy staff (two within the department and one outside the institute). These lesson plans were used while discussing clinical correlations of the fractures and visualizing the 3D models for the intervention group. On the other hand, the control group had a verbal discussion of clinical correlations without visualizing the models.

### Feedback and assessment

Kirkpatrick model levels I and II were assessed for the learning sessions [22].

At the end of the second session, each student in the interventional group was asked about their learning experience through a 5-point Likert scale questionnaire (Kirkpatrick model- level 1).

At the end of the second session, students’ knowledge and clinical reasoning for the topic were evaluated through an MCQ-based test. The clinical case-based questions focused on the anatomical structures at risk during fractures (Kirkpatrick model- level 2). The MCQs are provided as Additional File 2.

The feedback questionnaire and the MCQs were validated independently by three anatomy experts.

The test scores and the test-taking time were then compared between the groups. In order to maintain consistency in the educational benefits provided, the 3D-printed models were presented to the students in the control group alongside the remaining participants after the completion of the assessment. Subsequently, a discussion was conducted to explore the clinical significance of the models.

### Statistical analysis

The frequency and percentages were calculated for the learner satisfaction scale. The test scores and test-taking time between the control and the intervention groups were compared using an unpaired t-test. The open-ended responses were subjected to a qualitative analysis to identify the themes. We read and re-read the responses and identified the initial codes. We then combined the relevant codes and nested them into themes. We followed the step-by-step approach of doing thematic analysis given by Maguire and Delahunt [23]. SPSS version 16 was used for the statistical analysis (IBM Corp. Released 2016. IBM SPSS Statistics for Windows, Version 24.0. Armonk, NY: IBM Corp.).

## Results

The intervention group had 53 students, of which 20 were male and 33 were female. The control group had 52 students, of which 24 were male and 28 were female. The age of students ranged ranged between 18 and 22 years.

### Comparison of the test scores

Table 1 details the end-of-session test scores of the two groups. A significant difference was found between the two groups’ scores for the osteology quiz (*p* = 0.0151).

**Table 1:** Comparison of the test scores between the groups

	Intervention Group (*n* = 53)	Control Group (*n* = 52)
Mean and Standard Deviation	2.54 ± 1.15	2.04 ± 0.94
Maximum Score	5	5
Minimum Score	1	1
Significance (*p* < 0.05 is considered significant)	0.015*	

*as calculated by Unpaired t-test

The intervention group students took 5.15 ± 2.52 min to answer the test, with a maximum of 11.08 min and a minimum of 1.47 min. The control group took 4.91 ± 1.37 min with a maximum of 7.63 min and a minimum of 2.4 min. Both groups’ test-taking times were compared, and no significant difference was observed (*p* = 0.544).

### Student feedback

**Fig. 4:**
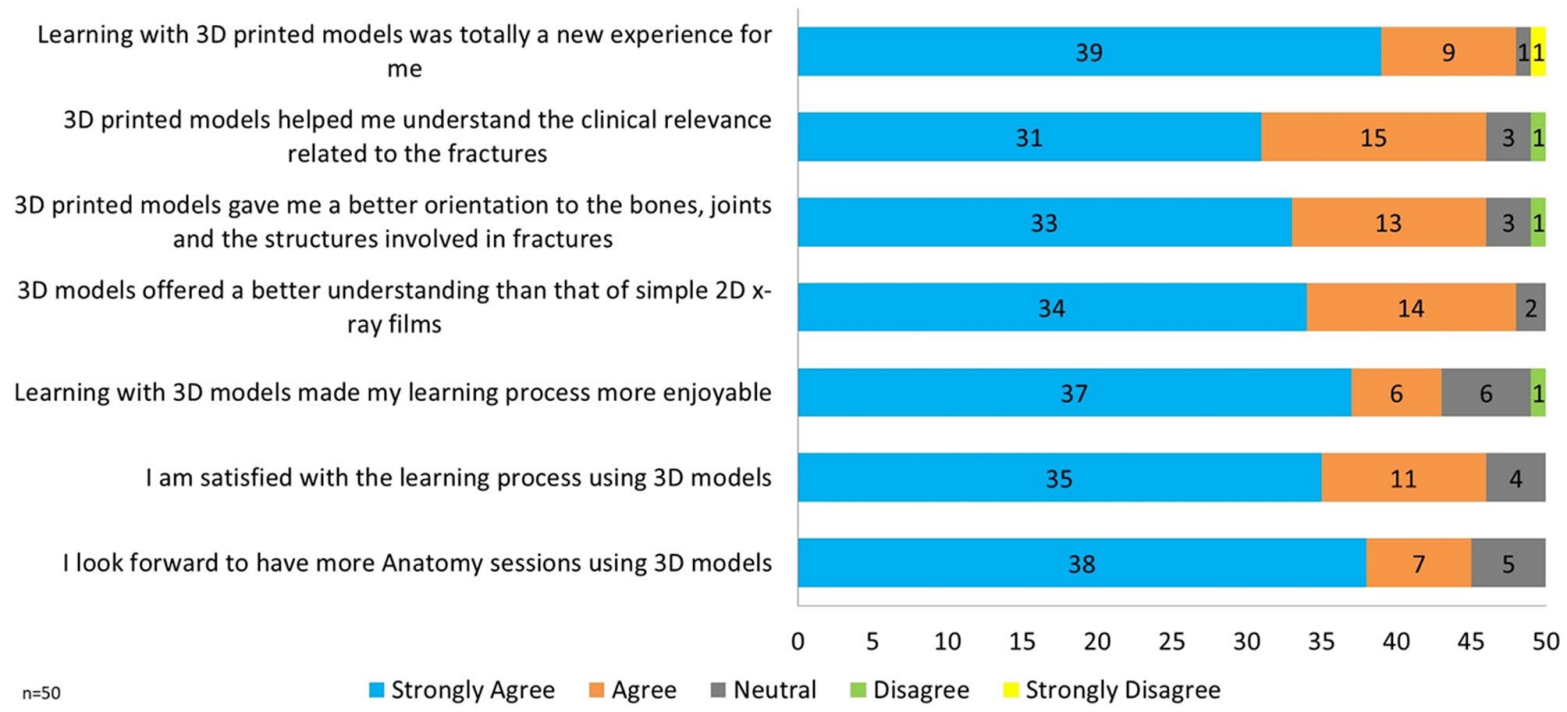
Frequency distribution of the student responses to the feedback questionnaire

We received feedback on the learning session from 50 intervention group students on a 5-point Likert Scale questionnaire (1 = Strongly Disagree and 5 = Strongly Agree). We excluded three students who did not fill out the feedback questionnaire. For 96% (*n* = 48) of the students, learning with 3D printed models was a new experience. 92% (*n* = 46) opined that 3D printed models helped them understand the clinical correlations of bones and gave them a better orientation to the bones and joints. 96% (*n* = 48) of students felt they could visualize and understand the fractures and the bones better than X-rays. 90% (*n* = 45) looked forward to more such sessions. A detailed view of the student feedback is given in Fig. 4.

### Thematic analysis of the responses

The thematic analysis of the open-ended questions revealed several themes. Table 2 provides a comprehensive compilation of the codes and themes.

**Table 2:** Thematic analysis of the open-ended responses displaying codes and themes

Questions	Codes	Themes
What did you like the most while using 3D-printed models for SGT classes?	Better understanding	Understanding the concept better
	Exciting and better understanding	
	Easy to clarify the concepts	
	Better orientation	Visual and kinaesthetic learning
	Easy to visualize the concept	
	Could feel the fractures	
	Bright and not dull like cadaveric specimens	
	Close to life-like structures	
	Better visualization	
	More interactive and visual	
	Helped in imagining and visualizing structures	
	Clear imagination of the problem/fracture	
	Fun and nice experience	Enjoyable learning experience
	An interesting part of SGT class	
	New experience and better understanding	
What did not go well while using 3D-printed models for SGT classes?	Less variety	Demand for more models
	Print more models for all bones	
	One model was not sufficient for a group of students	Dissatisfaction with the model-to-student ratio
	Time given per student was less	
	Few models were not clear	Challenges in visualization
	Fracture was not clearly visualized	
	Grooves and impressions were not as clear as real bones	
How can this strategy be taken forward?	Print more models for bones	Suggestions for improvement
	Add more variety	
	Better and larger models	
	Coloured models	
	Smaller student group	
	More such classes	
	Print models for all bones	

Three themes were identified for the question, ‘What did you like the most in the session?’. One theme was ‘understanding the concept better.’ One of the female students said, “Learning with 3D models was really exciting and new for me” (Student 12). Another female student said, “Orientation to the bones, joints, and structures involved helped me understand better.” (Student 35).

The second theme was ‘visual and kinaesthetic learning.’ One female student stated, “The proper understanding that’s impossible with 2D models was the most fun.” (Student 34). A male student responded, “We could actually feel the fractures and visualize them.” (Student 11).

The third theme was ‘enjoyable learning experience.’ A female student stated that students had a good learning experience: “It was a new experience overall, with better learning opportunities.” (Student 36).

Three themes were identified for the question ‘What did not go well?’. Those were; ‘demand for more models,’ ‘dissatisfaction with the model-student ratio,’ and ‘challenges in visualization.’ One male student responded, “Only one model per small group [20]; the model was smaller than normal and distorted in some cases.” (Student 22). “3D models should be made for all the bones.” (Student 25). Another male student said, “The grooves and different processes were not as well differentiated as in actual bone.” (Student 47).

For the question of how this strategy can be taken forward, we identified the theme ‘suggestions for improvement.’ Students wished to have more models, colored models of all bones in larger sizes. They also suggested having smaller student groups for better visualization.

## Discussion

The present study compares the utilization of 3D-printed models in osteology learning to traditional teaching in imparting clinical reasoning skills among undergraduate medical students. Our findings demonstrated that osteology teaching using 3D printed models could impact their understanding and clinical reasoning, as reflected by the higher test scores.

With the advancement of technology, 3D printed models serve as a valuable and reliable learning resource adjunct to wet cadaveric or plastinated specimens [24]. Especially the bones with complex anatomy, such as the skull, separate temporal bone, orbit, and paranasal sinuses, have been the choices of 3D printing, and their effectiveness in anatomy learning has been evaluated previously [12, 25–27]. Incorporating 3D-printed models of bones facilitates learning, especially structure recognition, as opined by Chen et al., which analyzed the immediate education effect examination using various question types [12]. Similarly, another study by Garas et al. compared the effectiveness of wet, plastinated, and 3D-printed models in identifying anatomical structures and revealed a larger proportion of students identifying structures using 3D-printed models [28]. In the present study, we utilized the 3D-printed models to analyze their effectiveness in assessing the higher domain of Bloom’s taxonomy in terms of clinical correlations (application and analysis levels), and the students of the intervention group performed significantly better, as reflected by their test scores.

3D-printed models could be developed based on educational needs, especially in clinical skill education, while acquiring new skills or practicing surgical skills [16]. These models offer a special relationship when a complex understanding is required, as opined by research conducted by Smerling et al., which used 3D-printed models of congenital heart diseases for teaching medical undergraduates [29]. The spatial anatomy of bones and understanding of fractures could be enhanced by using 3D-printed models of complex bones such as the pelvis and spine, as described by Wu et al. through their research [30]. In the present study, the students who saw the fractures in three dimensions better analyzed the case-based questions than those who had a verbal discussion.

Cultivating clinical reasoning skills in anatomy education is essential for the successful practice of medicine and provides a foundation for integrating basic sciences with clinical education [7, 31]. Case-based clinical reasoning (CBCR) is one of the approaches to teaching clinical reasoning to preclinical students, and it is ideally facilitated in small groups using the clinical problems they encounter in clinical practice [32]. A clinical case-based discussion focusing on the application of knowledge promotes interactive and deep learning [8]. Jiang et al. showed increased clinical reasoning skills by combining problem-based learning with 3D-printed models in neurosurgery education [33]. Similarly, a 3D-printed simulator effectively impacted clinical reasoning for orthodontic treatment planning among dental surgeons [34]. We have used a similar approach in the present study, incorporating the commonly encountered limb fractures to discuss their presentation and consequences on anatomic structures during the osteology classes.

In general, the students greatly appreciate any new approach to teaching. However, educational interventions need an elaborative description to be reproducible [35]. Similar results were obtained in the current study, with many students having enjoyable learning experiences and a better understanding of the clinical correlations related to fractures they visualized using the models. However, the end-of-session test compared the groups to analyze the educational benefit of the intervention.

To the best of the authors’ knowledge, this is the first attempt in India to incorporate 3D printed models in improving undergraduate medical students’ clinical reasoning abilities. Studies must focus on utilizing such models to retain skills for longer and how they can be applied in patient care while managing clinical conditions.

As a way forward, multicentre studies with a larger sample size would be required to generalize the findings. Additionally, comparing the effectiveness of physical 3D-printed models with digital 3D applications would offer insights into their applicability in advancing clinical education. Aligning the topics of assessment with the teaching is also a significant consideration when evaluating the effectiveness of these learning strategies.

### Limitations

Due to the time constraint in printing, the models were made smaller than the actual size, which made it challenging to identify the minor details. The test was conducted immediately after the learning session, and therefore, the results would be influenced by the immediate recall of the content discussed. However, it was addressed to a certain extent by framing the case-based questions of the higher domain of Bloom’s Taxonomy that required logical knowledge application. Though the students were taught using both upper and lower limb bones, the MCQ test focussed only on the upper limb bones. A minimal number of questions in the test also poses a challenge in comparing the scores for significance. Therefore, an extensive approach to teaching and assessing such an intervention with more case-based questions would be required.

## Conclusion

Incorporating 3D-printed models of fractures was a novel approach to help students comprehend the clinical correlations. This strategy improved students’ clinical reasoning skills in the intervention group, as evidenced by their higher scores. The students provided positive feedback on utilizing such models during routine osteology classes. Therefore, 3D-printed models are a valuable addition to the traditional teaching methods used in anatomy education. This could be an effective strategy to impart clinical reasoning skills during early clinical exposure to medical undergraduate students. As a way forward, future studies can focus on utilizing 3D-printed fracture models of all bones in actual sizes for osteology education with small student groups for better learning outcomes.

## Electronic supplementary material

Below is the link to the electronic supplementary material.

Supplementary Material 1

Supplementary Material 2

## Data Availability

No datasets were generated or analysed during the current study.

## Abbreviations

IEC: Institutional Ethics Committee
FDM: Fused Deposition Modeling
SGT: Small Group Teaching
KMC: Kasturba Medical College
STL: Stereolithography
ABS: Acrylonitrile Butadiene Styrene
CBCR: Case-based Clinical Reasoning

## References

[1] Estai M, Bunt S. Best teaching practices in anatomy education: a critical review. Ann Anat. 2016;208:151–7. 26996541 10.1016/j.aanat.2016.02.010

[2] Ghosh SK. Cadaveric dissection as an educational tool for anatomical sciences in the 21st century. Anat Sci Educ. 2017;10(3):286–99. 27574911 10.1002/ase.1649

[3] Viswasom AA, Jobby A. Effectiveness of Video demonstration over conventional methods in teaching osteology in anatomy. J Clin Diagn Res. 2017;11(2):JC09–11. 10.7860/JCDR/2017/24029.9429 PMC5376855 28384890

[4] Virtual laboratories. Using Pedestal 3D to Teach Forensic Osteology [Internet]. [cited 2024 Apr 18]. Available from: https://symposium.une.edu.au/presentation/virtual-laboratories-using-pedestal-3d-to-teach-forensic-osteology/

[5] Shi J, Fu S, Cavagnaro MJ, Xu S, Zhao M. 3D Printing improve the effectiveness of fracture teaching and medical learning: a Comprehensive Scientometric Assessment and Future perspectives. Front Physiol. 2021;12:726591. 35002749 10.3389/fphys.2021.726591 PMC8740219

[6] Early Clinical Exposure-MBBS-2019 [Internet]. 2019 [cited 2024 Apr 18]. Available from: https://www.nmc.org.in/wp-content/uploads/2020/08/Early_Clinical_Exposure-MBBS-07.08.2019.pdf

[7] Anderson M, Hills-Meyer PR, Stamm JM, Brown K. Integrating clinical reasoning skills in a pre-professional undergraduate human anatomy course. Anat Sci Educ. 2022;15(2):304–16. 33387378 10.1002/ase.2050

[8] Klement BJ, Paulsen DF, Wineski LE. Clinical correlations as a Tool in Basic Science Medical Education. J Med Educ Curric Dev. 2016;3:JMECD.S18919. 10.4137/JMECD.S18919 PMC5758745 29349328

[9] Armstrong P, Vanderbilt U. 2010 [cited 2024 Apr 29]. Bloom’s Taxonomy. Available from: https://cft.vanderbilt.edu/guides-sub-pages/blooms-taxonomy/

[10] Yuen J. What is the role of 3D Printing in undergraduate anatomy education? A scoping review of current literature and recommendations. MedSciEduc. 2020;30(3):1321–9. 10.1007/s40670-020-00990-5 PMC8368521 34457795

[11] Erolin C. Interactive 3D Digital Models for Anatomy and Medical Education. Adv Exp Med Biol. 2019;1138:1–16. 31313254 10.1007/978-3-030-14227-8_1

[12] Chen S, Pan Z, Wu Y, Gu Z, Li M, Liang Z, et al. The role of three-dimensional printed models of skull in anatomy education: a randomized controlled trail. Sci Rep. 2017;7(1):575. 28373643 10.1038/s41598-017-00647-1 PMC5428829

[13] Backhouse S, Taylor D, Armitage JA. Is this mine to keep? Three-dimensional Printing enables active, personalized learning in anatomy. Anat Sci Educ. 2019;12(5):518–28. 30406975 10.1002/ase.1840

[14] Jones DG. Three-dimensional Printing in anatomy education: assessing potential ethical dimensions. Anat Sci Educ. 2019;12(4):435–43. 30554454 10.1002/ase.1851

[15] Jones DG. Anatomists’ uses of human skeletons: ethical issues associated with the India bone trade and anonymized archival collections. Anat Sci Educ. 2023;16(4):610–7. 37039309 10.1002/ase.2280

[16] Maglara E, Angelis S, Solia E, Apostolopoulos AP, Tsakotos G, Vlasis K, et al. Three-dimensional (3D) Printing in Orthopedics Education. J Long Term Eff Med Implants. 2020;30(4):255–8. 33463925 10.1615/JLongTermEffMedImplants.2020036911

[17] Lim PK, Stephenson GS, Keown TW, Byrne C, Lin CC, Marecek GS, et al. Use of 3D printed models in Resident Education for the classification of Acetabulum fractures. J Surg Educ. 2018;75(6):1679–84. 29929817 10.1016/j.jsurg.2018.04.019 PMC6346736

[18] Li Z, Li Z, Xu R, Li M, Li J, Liu Y, et al. Three-dimensional printing models improve understanding of spinal fracture–A randomized controlled study in China. Sci Rep. 2015;5:11570. 26099838 10.1038/srep11570 PMC4477328

[19] Huang Z, Song W, Zhang Y, Zhang Q, Zhou D, Zhou X, et al. Three-dimensional printing model improves morphological understanding in acetabular fracture learning: a multicenter, randomized, controlled study. PLoS ONE. 2018;13(1):e0191328. 29342198 10.1371/journal.pone.0191328 PMC5771611

[20] Cook DA, Beckman TJ. Reflections on experimental research in medical education. Adv Health Sci Educ. 2010;15(3):455–64. 10.1007/s10459-008-9117-3 18427941

[21] Competency Based Undergraduate Curriculum for the Indian Medical Graduate [Internet]. 2018. Available from: https://www.nmc.org.in/wp-content/uploads/2020/01/UG-Curriculum-Vol-I.pdf 33864453

[22] Kirkpatrick Partners. LLC. [Internet]. [cited 2024 Apr 22]. The Kirkpatrick Model. Available from: https://www.kirkpatrickpartners.com/the-kirkpatrick-model/

[23] Maguire M, Delahunt B. Doing a thematic analysis: a practical, step-by-step guide for learning and teaching scholars. All Irel J High Educ. 2017;9(3):3351–33514.

[24] Mogali SR, Yeong WY, Tan HKJ, Tan GJS, Abrahams PH, Zary N, et al. Evaluation by medical students of the educational value of multi-material and multi-colored three-dimensional printed models of the upper limb for anatomical education. Anat Sci Educ. 2018;11(1):54–64. 28544582 10.1002/ase.1703

[25] Low CM, Choby G, Viozzi M, Morris JM. Construction of three-dimensional printed anatomic models for frontal sinus education. Neuroradiol J. 2020;33(1):80–4. 31081452 10.1177/1971400919849781 PMC7005993

[26] Shen Z, Yao Y, Xie Y, Guo C, Shang X, Dong X, et al. The process of 3D printed skull models for anatomy education. Comput Assist Surg. 2019;24(sup1):121–30. 10.1080/24699322.2018.1560101 31012745

[27] Vandenbossche V, Valcke M, Willaert W, Audenaert E. From bones to bytes: do manipulable 3D models have added value in osteology education compared to static images? Med Educ. 2023;57(4):359–68. 36453018 10.1111/medu.14993

[28] Garas M, Vaccarezza M, Newland G, McVay-Doornbusch K, Hasani J. 3D-Printed specimens as a valuable tool in anatomy education: a pilot study. Annals Anat. 2018;219:57–64. 10.1016/j.aanat.2018.05.006 29883617

[29] Smerling J, Marboe CC, Lefkowitch JH, Pavlicova M, Bacha E, Einstein AJ, et al. Utility of 3D printed Cardiac models for Medical Student Education in congenital heart disease: across a spectrum of Disease Severity. Pediatr Cardiol. 2019;40(6):1258–65. 31240370 10.1007/s00246-019-02146-8

[30] Wu AM, Wang K, Wang JS, Chen CH, Yang XD, Ni WF, et al. The addition of 3D printed models to enhance the teaching and learning of bone spatial anatomy and fractures for undergraduate students: a randomized controlled study. Annals Translational Med. 2018;6(20):403–403. 10.21037/atm.2018.09.59 PMC6230865 30498730

[31] Elizondo-Omaña RE, López SG. The development of clinical reasoning skills: a major objective of the anatomy course. Anat Sci Educ. 2008;1(6):267–8. 19109857 10.1002/ase.57

[32] ten Cate O, Custers EJFM, Durning SJ, editors. Principles and Practice of Case-based Clinical Reasoning Education: A Method for Preclinical Students [Internet]. Cham (CH): Springer; 2018. PMID: 31314234. 31314234

[33] Jiang W, Jiang W, Jin P, Zhang J, Xia J, Wei W, et al. Application of 3D printing technology combined with PBL teaching method in clinical teaching of cerebrovascular disease: an observational study. Med (Baltim). 2022;101(47):e31970. 10.1097/MD.0000000000031970 PMC9704919 36451448

[34] Lai CSH, Gan MJS, Yen CC, Foong KWC. Does 3D simulation impact clinical reasoning for orthodontic treatment planning of impacted maxillary canines? Seminars in Orthodontics. 2024 Jun 27. 10.1053/j.sodo.2024.06.011

[35] Olson CA, Bakken LL. Evaluations of Educational interventions: getting them published and increasing their impact. J Continuing Educ Health Professions. 2013 Spring;33(2):77. 10.1002/chp.21168 23775907

