# Supplementary material for: Introducing 3D printed models of fractures in osteology learning improves clinical reasoning skills among first-year medical students: a pilot study
Source: BMC Med Educ. 2025 Feb 6;25:190. doi: 10.1186/s12909-025-06746-2 (PMC11800631; doi:10.1186/s12909-025-06746-2)
Supplement: Supplementary file 1 — Supplementary Material 1 [file 12909_2025_6746_MOESM1_ESM.docx]

**LESSON PLAN FOR USING 3D PRINTED MODELS IN OSTEOLOGY CLASS**

**MODEL USED:** Fracture upper end of femur (Subtrochanteric fracture)

**COMPETENCIES:** AN14.1 Identify the given bone, its side, important features, &

keep it in an anatomical position

AN14.2 Identify & describe the joints formed by the given bone

AN17.2 Describe the anatomical basis of complications of

Fracture of neck of femur

OR2.10 Describe and discuss the aetiopathogenesis, mechanism

of injury, clinical features, investigations, and principles

of management of fractures of the proximal femur

**LEARNING OBJECTIVES:** By the end of the session, the student must be able to

1. Identify the parts of the upper end of the femur
2. Identify the type of fracture in the 3D-printed model
3. Correlate complications of fractured neck of the femur to the anatomical structures involved

**LESSON PLAN: Duration- 15 minutes**

1. **Task for students-** The teacher/facilitator gives the model to the students and allows them to identify the bones and the fracture site

**Teacher’s guide-** Students identify/teacher facilitates the horizontal fracture line at the upper end of the femur (Subtrochanteric fracture). Also, identify the weakening of the heads of the femur (probably age-related changes)

1. **Task for students-** Comparing with a real bone (femur and hip bone), students will demonstrate how they articulate with each other. Then, it will be compared with the model to see how different it is during the fracture. The teacher facilitates the process.

**Teacher’s guide-** Students articulate the hipbone and femur to form the hip joint. Compare it with the fractured model and notice the united fracture at the upper end of the femur. Also, students can discuss the parts and muscle attachments at the upper end of the femur.

1. **Task for students-** Students will verbalize the complications of femur neck fracture and justify the reason for them. The teacher shall explain them in case of any queries from the students.

**Teacher’s guide-** Emphasise on the placement of the lower limb (shortened and laterally rotated) and avascular necrosis in case of intracapsular fractures.

**MODEL USED:** Fracture condyle of tibia

**COMPETENCIES:** AN14.1 Identify the given bone, its side, important features, &

keep it in an anatomical position

AN14.2 Identify & describe joints formed by the given bone

AN18.6 Describe knee joint injuries with its applied anatomy

OR2.11 Describe and discuss the aetiopathogenesis, mechanism

of injury, clinical features, investigations, and principles of

management of (a) Fracture patella (b) Fracture distal

femur (c) Fracture proximal tibia with special focus on

neurovascular injury and compartment syndrome

**LEARNING OBJECTIVES:** By the end of the session, the student must be able to

1. Identify the parts of the upper end of the tibia
2. Identify type of fracture in the 3D-printed model
3. Correlate the complications with the anatomic structures damaged

**LESSON PLAN: Duration- 15 minutes**

1. **Task for students-** The teacher/facilitator gives the model to the students and allows them to identify the bones, and exact fracture site

**Teacher’s guide-** Students identify the fracture of medial condyle of the tibia. Notices the displacement of the two fractured segments.

1. **Task for students-** Comparing with a real bone (femur and tibia), students will demonstrate how they articulate with each other. Then compare it with the model how different it is during the fracture. The teacher facilitates the process.

**Teacher’s guide-** Students articulate femur and tibia to form the knee joint and compare it with the fractured model. They shall identify the parts and the site of attachments of ligaments that are prone to damage in such fracture (e.g. Medial meniscus, medial collateral ligament, ACL- Unhappy triad). Also, emphasise on the muscle attachments at the lower end of femur and upper end of the tibia.

1. **Task for students-** Students will verbalize the complications of medial condylar tibia fracture and justify the reason for them. The teacher shall explain them in case of any query from the students.

**Teacher’s guide-** Discuss unhappy triad.
