# Supplementary material for: Introducing 3D printed models of fractures in osteology learning improves clinical reasoning skills among first-year medical students: a pilot study
Source: BMC Med Educ. 2025 Feb 6;25:190. doi: 10.1186/s12909-025-06746-2 (PMC11800631; doi:10.1186/s12909-025-06746-2)
Supplement: Supplementary file 2 — Supplementary Material 2 [file 12909_2025_6746_MOESM2_ESM.docx]

1. A 27-year-old male painter is admitted to the hospital after falling from a ladder. Physical examination reveals that the patient is unable to abduct his arm more than 15 **°** and cannot rotate the arm laterally. A radiographic examination reveals an oblique fracture of the humerus. He has associated sensory loss over the shoulder area. Fracture of which of the following structures will most likely correspond to the symptoms of the physical examination?

**A.** Medial epicondyle

**B.** Glenoid fossa

**C.** Surgical neck of the humerus*

**D.** Middle third of the humerus

2. A 29-year-old female is examined in the emergency department after falling from her balcony. Radiographic examination reveals that she has suffered a broken clavicle, with associated internal bleeding. Which of the following vessels is most likely to be injured in clavicular fractures?

**A.** Subclavian artery

**B.** Cephalic vein

**C.** Internal thoracic artery

**D.** Subclavian vein*

3. A 31-year-old male hockey player fell on his elbow and is admitted to the emergency department. Radiographic examination reveals a fracture of the surgical neck of the humerus, producing an elevation and adduction of the distal fragment. Which of the following muscles would most likely cause the adduction of the distal fragment?

**A.** Brachialis

**B.** Teres minor

**C.** Pectoralis major*

**D.** Supraspinatus

4. Which of the following anatomic structures is primarily injured in case of a fracture shown in the picture?


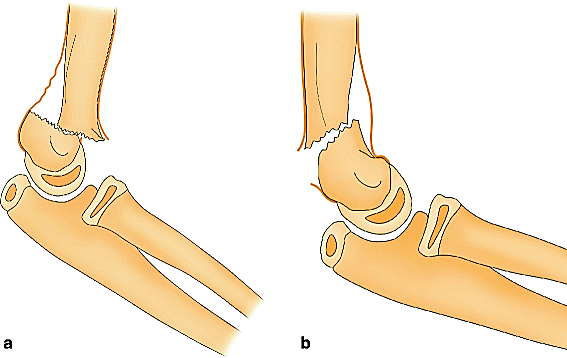


1. Ulnar nerve
2. Brachial artery*
3. Radial nerve
4. Profunda brachii artery

5. Which of the following muscles would produce an upward pull on the fractured segment denoted (arrowed) in the picture?


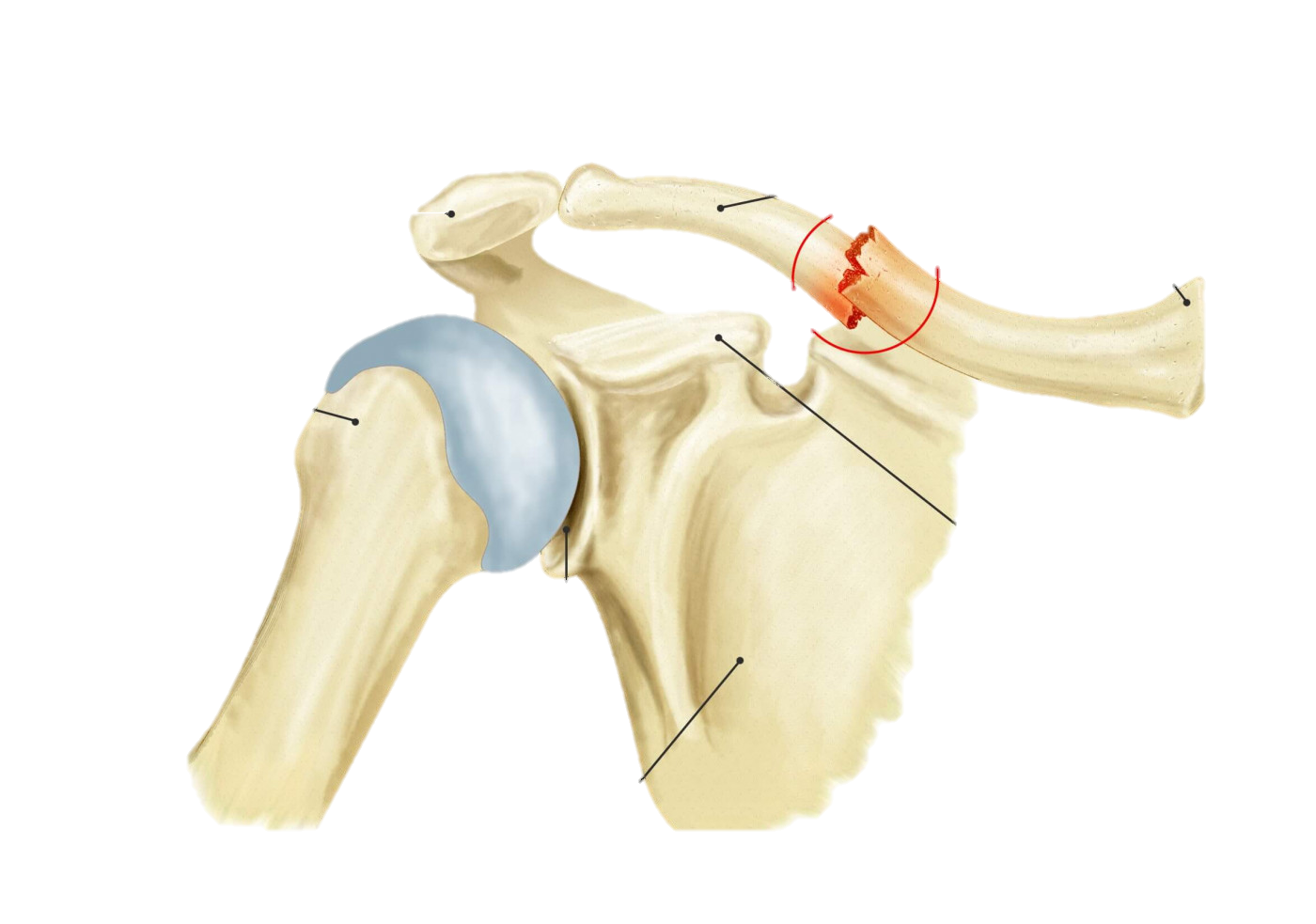


1. Sternocleidomastoid
2. Deltoid
3. Pectoralis major
4. Trapezius*
